# Direct and Indirect Roles of Macrophages in Hypertrophic Scar Formation

**DOI:** 10.3389/fphys.2019.01101

**Published:** 2019-08-28

**Authors:** Yi Feng, Zi-Li Sun, Si-Yu Liu, Jun-Jie Wu, Bin-Hong Zhao, Guo-Zhong Lv, Yong Du, Shun Yu, Ming-Lie Yang, Feng-Lai Yuan, Xiao-Jin Zhou

**Affiliations:** ^1^Department of Burns and Plastic Surgery, The Third Affiliated Hospital of Nantong University, Wuxi, China; ^2^Department of Pharmacy, Medical College, Yangzhou University, Yangzhou, China; ^3^Wuxi Clinical Medicine School of Integrated Chinese and Western Medicine, Nanjing University of Chinese Medicine, Wuxi, China

**Keywords:** hypertrophic scars, macrophages, wound healing, myofibroblast, differentiation

## Abstract

Hypertrophic scars are pathological scars that result from abnormal responses to trauma, and could cause serious functional and cosmetic disability. To date, no optimal treatment method has been established. A variety of cell types are involved in hypertrophic scar formation after wound healing, but the underlying molecular mechanisms and cellular origins of hypertrophic scars are not fully understood. Macrophages are major effector cells in the immune response after tissue injury that orchestrates the process of wound healing. Depending on the local microenvironment, macrophages undergo marked phenotypic and functional changes at different stages during scar pathogenesis. This review intends to summarize the direct and indirect roles of macrophages during hypertrophic scar formation. The *in vivo* depletion of macrophages or blocking their signaling reduces scar formation in experimental models, thereby establishing macrophages as positive regulatory cells in the skin scar formation. In the future, a significant amount of attention should be given to molecular and cellular mechanisms that cause the phenotypic switch of wound macrophages, which may provide novel therapeutic targets for hypertrophic scars.

## Introduction

Wound healing is a highly complex progressive process that involves intricate regulation and communication between multiple cell types (Steiling and Werner, 2003; Werner and Grose, 2003). It is made up of three successive phases, which are the inflammatory phase, proliferative phase, and remodeling phase (Wang et al., 2018). The final remodeling phase may result in hypertrophic scar or keloid formation in the dermis layer with excess collagen deposition, and the invasive growth of fibroblasts with the lack of cutaneous fat and hair follicles (Plikus et al., 2017). Abnormal scarring, such as hypertrophic scars and keloids, results in a remarkable alteration in appearance and function, and impairs the patient’s quality of life, both physically and psychologically (Van Loey and Van Son, 2003; Finnerty et al., 2016). It has been shown that genetic predisposition and skin injury play an important role in the formation of hypertrophic scars and keloids (Yuan et al., 2019). Different from hypertrophic scar, keloids are wounding-induced fibroproliferative tumor-like human scars. Keloids are more common in patients with darker skin types, with a prevalence of 4.5–16% in black and Hispanic populations. While the incidence of hypertrophic scars is reportedly higher than keloids in white people, ranging from 5 to 37% (Kose and Waseem, 2008). To date, there are no satisfactory preventive or therapeutic options for hypertrophic scars and keloids, and this is mainly due to the incomplete understanding of the underlying mechanisms (Mokos et al., 2017). Thus, it is extremely important to fully understand the regulatory mechanisms of hypertrophic scar and keloids formation and the controlled physiological process, including its pathophysiology, prevention and treatment.

Macrophages, which are produced from the bone marrow, circulate in peripheral blood or migrate to almost every tissue, and constitute the foremost controllers of both human innate and acquired immunity (Mosser and Edwards, 2008). Interestingly, macrophages are critical players in wound healing, providing pivotal signaling molecules for wound healing and coordinating wound healing processes. Macrophage dysfunction is characterized by an increase in the deposition of type I and III collagen, and myofibroblasts activation, which can impair the proper regenerative process, and otherwise, promote the development of fibrosis (Wei et al., 1999). Emerging evidence indicates that macrophages are essential for mounting either pro-fibrotic or anti-fibrotic responses at different stages during fibrotic pathogenesis (Clozel and Salloukh, 2005).

The present study summarizes the direct and indirect regulating roles of macrophages in skin wound healing and abnormal scar formation. Particularly, we emphasize that the significant direct effect of macrophages in scar formation was through its direct manipulation of the final ECM composition by secreting matrix metalloproteinases (MMPs) or its influence in producing collagen when they differentiate into myofibroblasts. In addition, the indirect effect on the activation and stimulation of myofibroblast leads to collagen deposition, thereby contributing to scar formation (Koh and DiPietro, 2011).

## Macrophage Lineage and Phenotypic Changes

Macrophages can be classified as resident tissue macrophages and monocyte-derived macrophages (Davies and Taylor, 2015). Monocytes, macrophages and dendritic cells (DCs) originate from dendritic cell progenitor cells (MDPs) in the post-partum stage, and monocytes are able to differentiate into DCs or macrophages in peripheral tissue sites (Geissmann et al., 2010). Activated DCs migrate to the lymph nodes where they present the antigen to immunocompetent T cells, in order to initiate an adaptive immune response (Iwasaki and Medzhitov, 2015). In contrast, macrophages largely remain in peripheral tissues after activation with their tissue-specific functions. Macrophages are antigen presenting cells and effectors of the elimination of Fc gamma receptor (FcγR)-dependent cells, and antibody-dependent anti-tumor responses mediate immune response (DiLillo and Ravetch, 2015).

Based on the expression of specific cell surface markers and their functional activation status, macrophages can be classified into two distinct populations. On one hand, macrophages that can be induced by interferon-γ (IFN-γ) and/or microbial components are referred to as M1 macrophages, which create a pro-inflammatory response that in return produce pro-inflammatory cytokines and chemokines, such as interleukin-6 (IL-6), IL-12, tumor necrosis factor-α (TNF-α), and CC chemokine ligand 2 (CCL2). These cytokines are an indispensable part of the initial process of wound healing. On the other hand, M2 macrophage-phenotypes are activated by IL-4 and/or IL-13, and characterized by secreting anti-inflammatory effectors, such as IL-10, transforming growth factor β1 (TGF-β1), heme oxygenase-1 (HO-1), and arginase. Through this, M2 macrophages regulate inflammatory responses, and participate in the control of wound healing and tissue regeneration (Tu et al., 2014).

The morphology and phenotypic evolution of macrophages are driven by various stimuli and cytokines from the environment (Mantovani and Sica, 2010). For example, in an infectious microenvironment, macrophages can be polarized into the “M1” state, and in cancerous tissues, there are different clues that induce “M2”-like properties (Solinas et al., 2010). There has been apparent controversy in literature on how the M2 phenotype is derived. Different subsets of wound macrophages can be derived from monocytes with different phenotypes recruited at different times. These monocytes could differentiate into macrophages with distinct phenotypes. During the course of wound healing, these recruited monocytes could be influenced by the constantly changing wound environment which could affect their polarization or cause the M1 macrophage differentiation into M2 macrophages (Martinez and Gordon, 2014). Multiple studies have agreed with the second hypothesis that it is the same macrophages that regulate early inflammatory functions and subsequent tissue regenerative functions (Wynn and Vannella, 2016). These observations suggest that the local tissue environment or some functions of macrophages can induce the phenotypic switch in the healing of wounds (Das et al., 2015). Thus, tracking macrophage phenotypes in the context of wound healing would be informative to individual patient, since it can provide an assessment of whether the skin wound exist within the context of a pro-inflammatory or anti-inflammatory environment, and this may provide guidance in developing targeted therapies (Ostuni et al., 2015). It is this contradictory view that makes macrophages such an attractive target for anti-scar therapies.

## The Direct and Indirect Roles of Macrophages During Scar Formation

Macrophages, which are known to play major role in tissue repair and regeneration, have drawn increasing attention to their potential roles in the development of scar formation. On one hand, a recent study revealed that M1 macrophages are mainly distributed in early wounds, while M2 macrophages are mainly distributed in late stage wounds and the proliferative phase of hypertrophic scars (Vogel et al., 2013). In skin wounds, M1 macrophage numbers peak at days 7–14, but the numbers of M2 macrophages in hypertrophic scar tissues increase at 14–28 days after wounding (Zhu Y. et al., 2016). On the other hand, macrophages exhibit distinct functions at various stages of skin wound repair (Minutti et al., 2017). If the chronic inflammatory phase of wound healing is prolonged, it might cause development of scar formation (Finnerty et al., 2016). Recently, Sinha et al. (2018) found that in the wound microenvironment macrophages directs fibroblasts proliferation, myofibroblast differentiation and collagen deposition. The changes in macrophage number and phenotype can disrupt wound healing process and determine the level of scar formation (Das et al., 2015). The extent of the inflammatory response is highly dependent on the polarization of macrophages into inflammatory M1 or anti-inflammatory M2 macrophages. The anti-inflammatory M2 macrophages regulate both the repair process and the final scar formation (Hesketh et al., 2017). A number of studies suggested that it is macrophages and related factors that regulate early inflammatory functions and later wound reparative functions (as summarized in Table 1). These findings have improved our understanding of the direct and indirect roles of diverse macrophage populations in tissue repair and scar formation.

**TABLE 1:** The role of macrophages and related factors in wound healing.

	**“Physiologic” wound healing**	**“Pathologic” wound healing**	**References (PMID)**
Main phenotype	M2	M1	Crane et al., 2014
Effect	anti-inflammatory	pro-inflammatory	Braga et al., 2015
Collagen	COLIII	COLI, COLIII, COLVIII	Nissinen and Kahari, 2015; Kreimendahl et al., 2019
Marker expression	CD206, CD163	CD86, CD80	Willenborg and Eming, 2014; Roszer, 2015
Cytokines	IL-10, TNF-α	IL-1β, IL-23, IL-6, TNF-α, IFN-γ	Butcher and Galkina, 2012
Proteases	MMP-1,13	MMP-7,10	Nissinen and Kahari, 2015; Amini-Nik et al., 2018
Signaling pathway	Wnt/β-catenin, TGF-β/Smad		Amini-Nik et al., 2014

*COL, collagen; IL, interleukin; MMP, matrix metalloproteinase; TNF-α, tumor necrosis factor α; IFN-γ, interferon-γ; TGF-β, transforming growth factor β.*

## The Direct Roles of Macrophages During Scar Formation

The direct role of macrophages in the formation of abnormal scar exhibits as its direct manipulation of the final ECM composition through the secretion of MMPs or its influence in producing collagen when these differentiate into myofibroblasts (Weitkamp et al., 1999). These are less well-known potential direct effects of macrophages, but they are the main reasons for abnormal scar formation, which suggests that macrophages could play a more decisive role in the wound healing process.

During the proliferation phase of the cutaneous wound healing, macrophages in the skin wound are more M2-like, and they function to increase the synthesis of ECM proteins. Increasing evidence suggests that macrophages are capable of synthesizing type VIII collagen, which is a short chain non-fibrillar collagen type, as demonstrated by Weitkamp et al. (1999). It is known that type VIII collagen adheres to the fibrosis by adhering to the ECM component, particularly type I collagen, thereby forming a coating on the type I collagen, facilitating the migration of the matrix and the binding to the skin fibrosis. Schnoor et al. (2008) revealed that in addition to synthesizing and secreting collagen VIII, macrophages can secrete almost all collagens, except for type XIII and XXII collagens. These findings suggest the direct role of macrophages in wound healing and scar formation. However, present studies on secreted collagen by macrophages associated with certain pathological conditions such as fibrosis have mainly focus on the heart and lungs (Vannella and Wynn, 2017). Therefore, an improved understanding of macrophages secreted ECM components in skin scar formation is urgently needed.

It has been shown that *in vitro*, cultured macrophages can differentiate into collagen-producing α-SMA myofibroblasts. Myofibroblasts are known as effector cells of scar formation (Lebonvallet et al., 2018). These cells are capable of synthesizing large amounts of ECM components, such as type I and III collagen, fibronectin, laminin, and other basal membrane proteins that are major constituents of scar tissues. Myofibroblasts are mainly differentiated from tissue-resident fibroblast, and they play a major role both in the scar process and in response to injury. However, myofibroblasts can also originate from other cells, such as macrophages, through which macrophages could directly contribute to collagen production, as transdifferentiated into myofibroblasts (Stone et al., 2016; Shook et al., 2018). Moreover, myofibroblasts are characterized by contractility and distorting structural organs, which are due to the expression of alpha-smooth muscle actin (α-SMA) (Rao et al., 2014). Other studies have also confirmed that M2 macrophages predominantly undergo macrophage-myofibroblast transition in chronic renal allograft injury (Wang et al., 2017). The majority of macrophage-to-myofibroblast transition cells were a major source of collagen-producing fibroblasts in the fibrosing kidney, accounting for more than 60% of α-SMA cells originating from macrophages (Wang et al., 2016). However, to what extent these myofibroblasts actually contribute to the total collagen production, particularly in scar formation, has not been completely elucidated.

**FIGURE 1:**
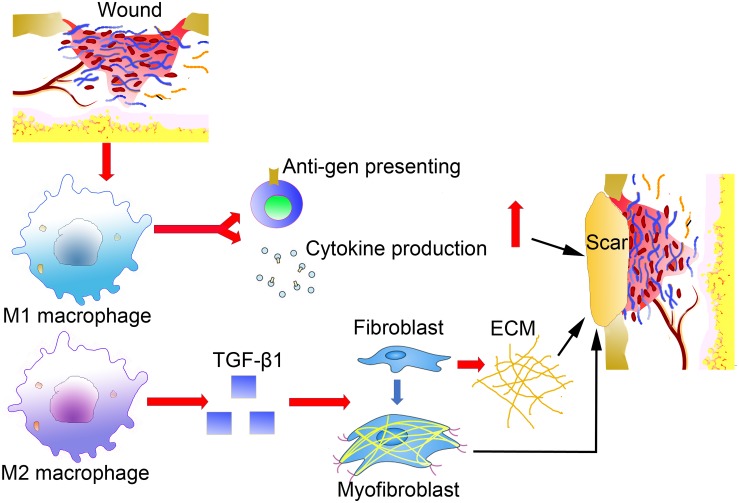
The direct effects of macrophages in abnormal scar formation.

Another important factor for the final hypertrophic scar formation and ECM components is the MMPs, which are secreted by almost all cell types in the wound environment. Specifically, macrophages can produce MMP-10 in response to skin injury in many tissues, including the skin (Koller et al., 2012). A recent study investigated the contribution of macrophage-derived MMP-10 during cutaneous wound healing in a mouse model (Rohani et al., 2015). Increased scar formation was observed in the wounded skin of MMP-10 -/- mice, but the number of macrophages in MMP-10 -/- mice did not decrease, and the mobility of macrophages was not impacted. However, in the skin wounds of MMP-10 -/- mice, the production and activity of MMP-13 produced by macrophages decreased, causing impaired scar resolution in the wounds. These results demonstrate the involvement of macrophages in skin scar formation and macrophage-derived MMPs in controlling tissue remodeling and alleviating scar formation during cutaneous wound repair (Figure 1). However, the exact role of the macrophage-derived MMPs in scar formation has not been fully explored at present.

## The Indirect Roles of Macrophages in the Formation of Abnormal Scar Formation

**FIGURE 2:**
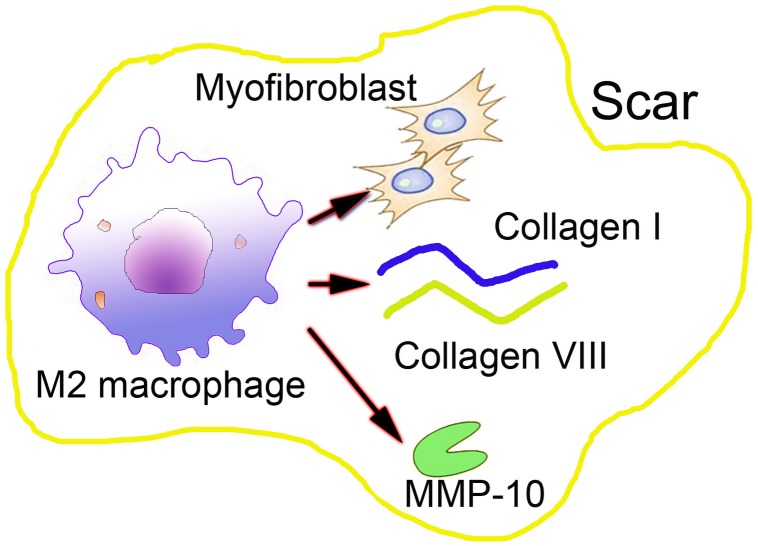
The indirect effects of macrophages in abnormal scar formation.

After blood coagulation and the formation of an early provisional matrix, macrophages are attracted to the wound area as the first responder. During the early phases of inflammation, M1-like macrophages have pro-inflammatory features, including cytokine production phagocytosis and antigen presenting (Eming et al., 2007). After the initial inflammatory response, macrophages also act a pivotal part in the successive proliferation, re-epithelialization and remodeling phases, which are not precisely defined by time, but by dynamic overlapping processes. Macrophages mostly differentiate into M1-like macrophages under the influence of pro-inflammatory mediators by IFN-γ and TNF (Novak and Koh, 2013). Chen et al. (2019) revealed that wound macrophages isolated in the early stage after wounding predominantly secrete M1-associated cytokines, such as TNF-α and IL-6, whereas those harvested from the long-term period of wound healing and hypertrophic scar formation produce more M2-associated cytokines, TGF-β1. Moreover, van den Broek et al. (2015) examined the dynamic changes of M2 macrophages in hypertrophic scars, and found that M2 macrophages increased during wound healing, arriving at the peak in the remodeling phase and decreasing during in the development of hypertrophic scars. However, as the injury continued to exist, M2 macrophages took part in promoting fibrosis and secreted factors, such as TGF-β, as detected in human post-burn hypertrophic scars, which could indirectly promote ECM production and fibroblast-to-myofibroblast differentiation (Kurose and Mangmool, 2016). The transcription of the TGF-β gene results in proliferation, contraction, ECM production, autocrine TGF-β secretion upregulation and differentiation in myofibroblasts (Walraven et al., 2014). These studies suggest that macrophages play an important role in scar formation by activating myofibroblasts through the secretion of TGF-β (Figure 2).

In contrast to previous limited studies, Jin et al. (2018) have demonstrated that macrophages were highly active in keloid tissues and were polarized toward the M2 macrophages phenotype. Moreover, these M2 macrophages in keloid tissues could induce regulatory T cells differentiation by upregulating Foxp3 expression (Jin et al., 2018). Furthermore, in keloid tissues, the number of M2 macrophage infiltration might be related to the sensitivity of the glucocorticoid receptor.

## Targeting Macrophages as Therapeutic Approaches in Hypertrophic Scar Treatment

Wound macrophages and macrophage-derived products participate in all phases of wound healing, and these macrophage-directed processes of tissue repair and fibrosis are shared among scar-associated diseases and affect the organs. Thus, macrophage-directed interventions provide an interesting strategy for preventing and treating pathological scarring (Smigiel and Parks, 2018). Indeed, recent evidence of effectively targeting macrophages in hypertrophic scar animal models positions macrophage as an attractive candidate for developing clinical therapeutic strategies against hypertrophic scars.

The systemic depletion of macrophages in a hypertrophic scar formation animal model, effectively inhibited hypertrophic scar formation in the subacute phase of wound healing, including collagen remodeling, mast cell infiltration, myofibroblast formation, and decreased pro-fibrotic factors. Moreover, macrophage depletion also down-regulated M1-related cytokines (TNF-α, IL-1β, and IL-6) and M2-related cytokines (TGF-β1, IL-10, and IL-1α) in grafted tissues (Zhu Z. et al., 2016). Likewise, the recent finding in a macrophage selectively depleted transgenic mice model suggested that macrophages have a key role in the early phases of skin wound repair. During the inflammatory phase, the depletion of macrophages was found to significantly decrease the formation of vascularized granulation tissues and impair epithelialization, which ultimately reduce the degree of granulation tissue and scar formation (Ellis et al., 2018). Another example related to the blockade of macrophage signaling pathways is the TGF-β signaling–deficient Smad3 knockout. The loss of Smad3 recruited macrophages into the skin wounds and healed without scarring (Ashcroft and Roberts, 2000).

## Conclusion

Collectively, a large body of evidence suggests that macrophages are major contributors to several pathomechanisms that lead to abnormal wound healing, such as fibrotic scars. Studies with the successful intervention of hypertrophic scars using the depletion of macrophages or blockade of macrophage signaling in various experimental animal models could serve as an important basis to further develop drugs aimed at attenuating or resolving hypertrophic scars. Macrophages are regarded as be a highly a heterogeneous cell population that can be developed from different sources. M1-like macrophages are considered foe cells associated with pro-inflammatory and pro-fibrotic functions. On the other hand, M2-like macrophages becomes friend of the wound healing. However, when the skin wound is not controlled and there is a continuous activity of M2-like macrophages, these cells play the part of an enemy for would healing and scar formation through direct or indirect effects (Braga et al., 2015). A thorough understanding of the direct and indirect roles of macrophages in would healing and scar formation, especially the molecular phenotype of these cells at different stages and in pathological wound healing, become further defined. New macrophage-based therapies for fibrodestructive disorders such as hypertrophic scar formation should emerge (Amini-Nik, 2018). Several strategies have been developed and adopted with the aim of manipulating macrophages, and macrophage reprogramming and macrophage depletion can be regarded as the most important methods.

## Author Contributions

F-LY, YF, and Z-LS contributed to the writing of the manuscript. S-YL, J-JW, B-HZ, G-ZL, YD, SY, M-LY, and X-JZ participated in the revision of the manuscript. X-JZ contributed to the concept of the article. S-YL, J-JW, and G-ZL were responsible for the production of pictures and forms. YD, SY, and M-LY contributed to the revision and improvement of the article.

## Conflict of Interest Statement

The authors declare that the research was conducted in the absence of any commercial or financial relationships that could be construed as a potential conflict of interest.
